# Supplementary material for: Readiness to prescribe: Using educational design to untie the Gordian Knot
Source: PLoS One. 2020 Jan 24;15(1):e0227865. doi: 10.1371/journal.pone.0227865 (PMC6980548; doi:10.1371/journal.pone.0227865)
Supplement: S1 Fig — (PDF) [file pone.0227865.s001.pdf]

smac[2]
Making Insulin Treatment Safer

Making Insulin Treatment Safer
Participant Information Sheet

Thank you for your interest in this project. Please read this information sheet carefully before deciding if you would like to participate. If you choose not to participate, we thank you for considering our request.

What is the purpose of the study?

Identify capabilities, opportunities, and motivations supporting safe insulin prescribing in the Northern Ireland Diabetes Education Community.

Why have I been invited to participate?

Because you are a pharmacist, foundation doctor, registrar/consultant, nurse, or leader who contributes to the system of care in which foundation doctors learn to prescribe insulin safely. We have developed a complementary set of questionnaires, individualised to those 5 professional groups.

Do I have to take part?

It is completely your choice whether or not to take part. Completing a questionnaire will be taken as consent. If you do not consent, please do not complete one.

What will happen to me if I take part?

You are asked to choose the number best describing your response to each item in the questionnaire. Please enter a free text response to any or all of the items if you want to say something you would like us to hear. Please do not name individuals or give any confidential information that would allow us to identify them. We are interested in the system, not the individuals who work in it.

When will this study take place?

As soon as you are ready to complete the questionnaire.

Where will the study take place?

Wherever you would like to complete it.

Will the information I give be confidential?

We do not wish to know the identities of individuals who complete the questionnaires and have not included any fields that could make you identifiable. Completed questionnaires will be stored securely at the University in a locked filing cabinet and/or on password protected computers. In accordance with current ethical guidelines, records will be kept for 5 years after publication.

During the time we keep the information, we may use it to teach other students how to analyse information of the sort you gave us. We may also wish to carry out further analysis of the information you gave, to answer additional research questions which may arise. In either eventuality, we will continue to keep the information entirely confidentially.

What happens if I want to complain about this research?

Should you wish to make a complaint, you may do so to the senior researcher, whose name is at the bottom of this consent form. He will arrange to meet you personally and respond to your dissatisfaction.

Please note the following points:

1. Your participation is entirely voluntary
2. You may decline to answer any question or discard the questionnaire at any point.
3. Your participation, or non-participation, will not affect your legal rights
4. The information collected will be treated securely and confidentially as necessary under the Data Protection Act and stored as required by the University.
5. All results, if published, will be treated anonymously
6. The results of the study, if published, may include direct quotes from your questionnaire but not your identity.
7. The results of MITS will be presented at local, national, and international meetings
8. You can contact Professor Tim Dornan, 02890 975773, t.dornan@qub.ac.uk with any concerns during the study

HSC Public Health Agency
Research and Development
QUEEN'S UNIVERSITY BELFAST

© 2017, The Queen's University of Belfast and HSCNI, all rights reserved. Funding was provided by HSCNI.

Making Insulin Treatment Safer (MITS) Junior Doctors' Readiness to Prescribe Well (RtoRx[jd])

Please answer all these questions in relation to prescribing insulin

Hospital: **Grade:** (and if relevant, speciality): **Sex:** Male ☐ Female ☐ Other / Prefer not to say ☐

Have you prescribed insulin? Yes ☐ No ☐

How often do you do so? Daily   Several times a week   Several times a month   Monthly   Less often than monthly

If you do prescribe insulin, please tick the statement that best describes you:

- I feel no need to learn to prescribe insulin better ☐
- I would like to learn to prescribe insulin better ☐
- I am actively learning to prescribe insulin better ☐

Please rate your agreement with the following statements **as they apply to insulin prescribing**. Circle a number from 0 (completely disagree) to 6 (completely agree). There are several places in the questionnaire where we invite you to add written comments. Whilst that is optional, please rate **all** the numerical items.

Your capability to prescribe insulin

Completely Disagree						Completely Agree
**1. I think out prescriptions logically rather than by habit**						
0	1	2	3	4	5	6
**2. I can distinguish simple prescribing decisions from difficult/ambiguous ones**						
0	1	2	3	4	5	6
**3. I can judge whether my knowledge and skills are sufficient for individual prescribing decisions**						
0	1	2	3	4	5	6
**4. When I recognize what action needs to be taken, I prescribe without hesitation**						
0	1	2	3	4	5	6
**5. I feel safe to put into practice what I learn about prescribing**						
0	1	2	3	4	5	6
**6. I am confident that I am on the path to being a good prescriber**						
0	1	2	3	4	5	6

Things that make me more capable to prescribe (as defined by those six statements) are:

Things that make me less capable to prescribe are:

© 2017, The Queen's University of Belfast and HSCNI, all rights reserved. Funding was provided by HSCNI.

Influences on your capability to prescribe insulin						
Completely Disagree						Completely Agree
7. Tensions with senior (or junior) doctors affect my capability to prescribe well						
0	1	2	3	4	5	6
8. Tensions with other health professionals (eg nurses/ pharmacists) affect my capability to prescribe well						
0	1	2	3	4	5	6
9. Other people's standards of prescribing affect my capability to prescribe well						
0	1	2	3	4	5	6
How other people (as defined by those three statements) influence my capability to prescribe for the better:						
How other people influence my capability to prescribe for the worse:						

Your capability to learn insulin prescribing 'on-the-job'						
Your behaviour and preferences:						
10. When I am unsure what is the right action, I seek guidance						
0	1	2	3	4	5	6
11. I use learning tools to increase my knowledge and skills						
0	1	2	3	4	5	6
12. I (would) like to receive constructively critical feedback on my prescriptions						
0	1	2	3	4	5	6
Your habits						
13. I am in the habit of consulting books/online resources/guidelines to help me prescribe						
0	1	2	3	4	5	6
14. I am in the habit of discussing prescriptions with other doctors (seniors or peers)						
0	1	2	3	4	5	6

© 2017, The Queen's University of Belfast and HSCNI, all rights reserved. Funding was provided by HSCNI.

15. I am in the habit of discussing prescriptions with nurses or pharmacists						
0	1	2	3	4	5	6

16. I am in the habit of involving patients in prescriptions						
0	1	2	3	4	5	6

Things that encourage me to learn on-the-job (as defined by those seven statements):

Things that discourage me from learning on-the-job:

Influences on your capability to learn insulin prescribing 'on-the-job'

17. The people where I work support my learning to prescribe						
0	1	2	3	4	5	6

18. The people where I work encourage/support me to reflect critically on the quality of my prescriptions						
0	1	2	3	4	5	6

19. The people where I work give credit for good prescribing						
0	1	2	3	4	5	6

20. The people where I work give me constructively critical feedback on my prescribing						
0	1	2	3	4	5	6

21. The people where I work make a virtue out of acknowledging uncertainty and seeking help						
0	1	2	3	4	5	6

22. Practically useful information about prescribing/prescribing aids are available to me						
0	1	2	3	4	5	6

How my on-the-job experiences (as defined by those six statements) influence my learning for the better:

How my on-the-job experiences influence my learning for the worse:

© 2017, The Queen's University of Belfast and HSCNI, all rights reserved. Funding was provided by HSCNI.

Your undergraduate and foundation education						
Completely Disagree						Completely Agree
**23. My prior education prepared me to prescribe during my foundation posts**						
0	1	2	3	4	5	6
**24. I expect my foundation education will result in me prescribing well**						
0	1	2	3	4	5	6
How my prior and/or present education are contributing:						
How my prior and/or present education are not contributing or could contribute more:						

Thank you for completing this questionnaire.

© 2017, The Queen's University of Belfast and HSCNI, all rights reserved. Funding was provided by HSCNI.
